# Primary Care Practice Telehealth Use and Low-Value Care Services

**DOI:** 10.1001/jamanetworkopen.2024.45436

**Published:** 2024-11-07

**Authors:** Terrence Liu, Ziwei Zhu, Michael P. Thompson, Jeffrey S. McCullough, Hechuan Hou, Chiang-Hua Chang, A. Mark Fendrick, Chad Ellimoottil

**Affiliations:** 1 Institute for Healthcare Policy and Innovation, Department of Internal Medicine, Veterans Affairs Center for Clinical Management Research (CCMR), Ann Arbor, Michigan; 2 University of Michigan, Ann Arbor; 3 Institute for Healthcare Policy and Innovation, Department of Urology, University of Michigan, Ann Arbor; 4 Institute for Healthcare Policy and Innovation, Department of Cardiac Surgery, University of Michigan, Ann Arbor; 5 Institute for Healthcare Policy and Innovation, Department of Health Management and Policy, University of Michigan, Ann Arbor; 6 Institute for Healthcare Policy and Innovation, Department of Internal Medicine, University of Michigan, Ann Arbor

## Abstract

**Importance:**

The rapid expansion of telehealth transformed how primary care practices deliver care; however, uncertainties about the quality of telehealth-delivered care compared with in-person care remain. While there are concerns that increased telehealth may introduce wasteful care, how telehealth affects the delivery of low-value care is unknown.

**Objective:**

To examine whether a primary care practice’s level of telehealth use is associated with changes in the rates of low-value care.

**Design, Setting, and Participants:**

This retrospective cohort study using a difference-in-differences study design was conducted from January 1, 2019, to December 31, 2022, using Medicare fee-for-service claims data. Participants were Medicare beneficiaries attributed to primary care practices in Michigan.

**Exposures:**

Low, medium, or high tertile of practice-level telehealth use.

**Main Outcomes and Measures:**

Low-value care was assessed using 8 claims-based measures relevant to primary care, grouped into 4 main categories: office-based, laboratory-based, imaging-based, and mixed-modality services. Poisson regression models were used to estimate the association between practice-level telehealth use and rates of low-value care services, controlling for practice-level characteristics.

**Results:**

A total of 577 928 beneficiaries (332 100 [57%] women; mean [SD] age, 76 [8] years) attributed to 2552 primary care practices were included in the study. After adjusting for practice-level characteristics and baseline differences in low-value care rates between telehealth use groups, high practice-level telehealth use was associated with lower rates of low-value cervical cancer screening (−2.9 [95% CI, −5.3 to −0.4] services per 1000 beneficiaries) and lower rates of low-value thyroid testing (−40 [95% CI, −70 to −9] tests per 1000 beneficiaries) compared with low practice-level telehealth use. Of the other 6 outcomes examined, there was no association between practice-level telehealth use and rates of low-value care services.

**Conclusions and Relevance:**

In this cohort study of Medicare fee-for-service beneficiaries who received care from primary care practices in Michigan, some low-value care services (ie, cervical cancer screening among women older than 65 years and low-value thyroid testing) were lower among practices with high telehealth use, and there was no association between practice-level telehealth use in rates of most other low-value care services not delivered in the office. As telehealth continues to be an important part of care delivery, evaluating how it may encourage or discourage low-value care services is critical to understanding its impact on quality of care.

## Key Points

**Question:**

Is practice-level telehealth use associated with the rates of low-value care services in primary care?

**Findings:**

In this cohort study with 577 928 participants, office-based low-value care services were lower among practices with high telehealth use, and there was no association between practice-level telehealth use and rates of most other low-value care services not delivered in the office.

**Meaning:**

Our findings suggest the potential for telehealth to help reduce office-based low-value care and could reassure policymakers concerned about telehealth encouraging unnecessary or wasteful care due to added convenience.

## Teaser

This cohort study evaluates whether the practice-level use of telehealth is associated with delivery of low-value care in primary care practices in Michigan.

## Introduction

Telehealth use has rapidly increased over the last several years and transformed the way outpatient care has been delivered by bridging geographic distances and increasing patients’ access to care. While the COVID-19 pandemic initially accelerated the widespread expansion of telehealth to facilitate social distancing, telehealth is likely to remain a permanent fixture of our current and future health care system. As policymakers decide how to finance and regulate telehealth going forward, concerns have been expressed that telehealth may generate wasteful or lower-quality care compared with traditional in-person care.^1,2,3,4,5^ Compared with in-person care, conducting a clinical encounter in a virtual setting may influence medical decision-making in ways that lower quality, such as clinicians overprescribing antibiotics.^6^ Given how integrated telehealth has become in our current health care system, evaluating its impact on cost and quality is critical to informing how health systems can best utilize telehealth effectively.

While previous studies evaluated the impact of telehealth on quality and costs of ambulatory care,^7,8,9,10^ how telehealth influences low-value care delivery is uncertain. Low-value care is defined as services that provide little to no clinical benefit for patients, have potential to cause harm, incur unnecessary cost, or waste limited health care resources.^11,12^ Telehealth has the potential to either increase or decrease low-value care. For example, virtual visits can eliminate opportunities for clinicians to perform low-value, in-person, office-based services, such as cervical cancer screening in women older than 65 years.^13^ On the other hand, the inability to conduct a physical examination may create more clinical uncertainty, leading to low-value diagnostic testing, such as imaging for uncomplicated low back pain. Low-value care has been extensively studied within Medicare using claims-based measures.^14,15,16,17,18^ Experts estimate the cost of waste from low-value care ranges from $75 to $101 billion each year.^19^ However, low-value care has not been studied in the context of telehealth.

In our study, we build on prior investigations of low-value care by examining the ways telehealth may influence its delivery. Specifically, we identified 4 categories of low-value care that may be differentially affected by telehealth use: office-based, laboratory-based, imaging-based, and mixed-modality services. We then examined practice-level telehealth use and how that may be associated with rates of low-value care services across these 4 areas. We hypothesized that high practice-level telehealth use would be associated with a decrease in office-based and laboratory-based low-value services and an increase in imaging-based low-value services, while mixed-modality low-value services would remain largely unaffected. Given the rapid expansion of telehealth, our study leverages a unique opportunity to investigate its association with rates of low-value care. The objective of our study is to enhance understanding of this association and to generate new insights that can guide policy decisions on telehealth.

## Methods

### Data and Study Population

This retrospective cohort study analyzed Medicare fee-for-service claims data from January 1, 2019, to December 31, 2022, using Part B Carrier and Outpatient Files. Access and use of Medicare claims data were approved and provided through a data use agreement between the University of Michigan and the Centers for Medicare & Medicaid Services (CMS) and the Michigan Value Collaborative. This study was determined to be exempt from review and the requirement for informed consent by the University of Michigan institutional review board because the data used in the study had been deidentified and coded. This study followed the Strengthening the Reporting of Observational Studies in Epidemiology (STROBE) reporting guideline.

Our cohort consisted of beneficiaries who were Michigan residents who were continuously enrolled during the year in which they received an outpatient evaluation and management service. Beneficiaries were attributed to primary care physicians and associated practices based on tax identification number using the CMS 2-step attribution method.^20^ Practices that had fewer than 10 attributed beneficiaries during the study period were excluded from the analyses. Practices that were extremely outliers (>99th percentile) in their telehealth use, defined later, were excluded.

### Defining Practice-Level Telehealth Use

To measure practice-level telehealth use, we stratified practices into weighted tertiles based on the number of telehealth services per 1000 beneficiaries used in 2022. We used data from 2022 to determine practice-level telehealth use because telehealth services represented a small fraction of overall evaluation and management services prior to the onset of the COVID-19 pandemic in 2020. We identified telehealth services using Medicare’s list of eligible telehealth services and the appropriate modifier codes (GT, GQ, and 95) or place of service code (02) corresponding to each year of the study. We also identified telehealth services using Healthcare Common Procedure Coding System codes for selected virtual care services including phone visits, virtual check-ins, online digital evaluations, interprofessional consultations, and remote monitoring^7,21^ (eTable 1 in Supplement 1).

### Practice-Level Characteristics

Practice-level characteristics were selected a priori to be included in our statistical model as covariates and included beneficiary count, number of in-person outpatient visits per 1000 beneficiaries, age (share of beneficiaries aged <65, 65-74, 75-84, ≥85 years), gender (share of men and women beneficiaries), race and ethnicity (share of beneficiaries in Asian, Black, Hispanic, White, and other [defined as categories not captured by Medicare race and ethnicity variables] categories), dual-eligibility (share of beneficiaries eligible for Medicaid), and rurality (defined at the zip code level based on the Department of Agriculture’s Rural-Urban Commuting Area Codes^22^). Race and ethnicity data are self-reported from the Medicare Beneficiary Summary File and were included to assess for telehealth-related health disparities. We performed risk adjustment using CMS Hierarchical Condition Categories (HCC) risk adjustment model to calculate average HCC risk score and its square, both of which were included in our statistical model.^8,9,23^

### Low-Value Care Measures

Our primary outcome was the difference in risk-adjusted rate of low-value care services between the prepandemic period (2019) and the postpandemic period (2022), comparing high- and medium-tertile primary care practices with low-tertile primary care practices. Outcomes are reported as the rate of low-value care services per 1000 eligible beneficiaries, estimated using average marginal effects (AMEs), which quantify the average change in the estimated outcome variable for a unit change in an explanatory variable, averaged over the entire sample of observations.^7^ We used well-established methods^14,15,16^ to calculate rates of 8 low-value care services relevant to primary care, applying specific criteria of low-value care that limits inclusion of appropriate use of services (eTable 2 in Supplement 1). We grouped these services into 4 main categories that may be affected by telehealth use in different ways: (1) office-based services (1 service), (2) laboratory-based services (3 services), (3) imaging-based services (3 services), and (4) mixed-modality services (1 service). Office-based low-value care services included cervical cancer screening for women older than 65 years. Laboratory-based low-value care services included prostate-specific antigen (PSA) testing for men older than 75 years, total or free T3 level testing for patients with hypothyroidism, and vitamin D testing in the absence of kidney disease or hypercalcemia. Imaging-based low-value care services included computed tomography (CT) of the sinuses for uncomplicated acute rhinosinusitis, head imaging for uncomplicated headache, and back imaging for patients with nonspecific low back pain. Because colorectal cancer screening can be performed either with laboratory-based tests (fecal immunochemical testing) or procedures (colonoscopy or flexible sigmoidoscopy), we categorized colorectal cancer screening for adults older than 85 years as mixed-modality low-value care services. These outcome measures were chosen to specifically target common low-value services within primary care that can be measured through Medicare claims data.^15^

### Statistical Analysis

We used the χ^2^ and Kruskal-Wallis tests to examine differences in telehealth adoption across practice-level characteristics. We used multivariable Poisson regression with a difference-in-differences design to estimate the association between telehealth use and change in rate of low-value care services between 2019 and 2022, comparing practices in high and medium tertiles of telehealth use to those in the lowest tertile of telehealth use. This association between practice-level telehealth use and the change in rate of low-value care services is represented by AMEs. Given that the different low-value care services have different inclusion and exclusion criteria for beneficiaries, we estimated 8 regression models for each low-value care service. In all models, we adjusted for practice-level characteristics and clustering at the practice level by calculating robust standard errors using the VCE command and cluster option in Stata version 18 (StataCorp). While our main analysis compared annualized rates of low-value care services in 2019 vs 2022, we assessed the parallel-trends assumption by performing our regression analysis of quarterly rates of low-value care services in the prepandemic period of 2019, estimating an interaction term between practice-level telehealth use and quarter. These estimates were largely statistically nonsignificant, suggesting that the parallel-trends assumption was upheld (eFigure in Supplement 1). We conducted 2-sided hypothesis tests with a significance level of α = .05. All analyses were performed in Stata version 18 (StataCorp) and SAS version 9.4 (SAS Institute).

## Results

### Practice-Level Characteristics

In our study, 577 928 beneficiaries (332 100 [57%] women; 8504 [1%] Asian, 37 802 [7%] Black, and 504 026 [87%] White individuals; mean [SD] age, 76 [8] years) were attributed to 2552 primary care practices in 2022. There was a greater mean (SD) percentage of beneficiaries living in rural areas among practices in the low tertile of telehealth use (43% [40]) compared with those in the medium (21% [32]) and high (16% [28]) tertiles of telehealth use in 2022 (Table 1). Other practice-level characteristics, including gender, age, race and ethnicity, and dual-eligibility, had similar distributions across practices in different tertiles of telehealth use (Table 1).

**Table 1.** Characteristics of Low-, Medium-, and High-Telehealth Use Primary Care Practices in 2019 and 2022

Characteristic	Practices by tertile of telehealth use, mean (SD)					
	Low (n = 1325)		Medium (n =580)		High (n = 647)	
	2019	2022	2019	2022	2019	2022
Beneficiaries per practice, No.	219 (541)	135 (436)	418 (1386)	366 (1352)	333 (1551)	288 (1451)
Visits per 1000 beneficiaries						
Telehealth	7 (29)	194 (155)	5 (39)	654 (116)	13 (115)	1469 (522)
In-person	10 200 (6568)	7862 (9664)	12 781 (6534)	11 670 (4921)	16 183 (11 822)	13 557 (7492)
Age, %^a^						
65-75	51 (15)	51 (17)	52 (14)	51 (15)	55 (13)	53 (14)
75-84	32 (8)	33 (11)	32 (8)	34 (10)	31 (8)	34 (10)
≥85	16 (12)	16 (14)	15 (10)	15 (10)	14 (9)	13 (9)
Gender, %^a^						
Women	58 (12)	55 (13)	60 (11)	57 (12)	60 (12)	59 (12)
Men	42 (12)	45 (13)	40 (11)	43 (12)	40 (12)	41 (12)
Race/ethnicity, %^a^						
Asian	1 (3)	1 (6)	2 (4)	2 (4)	3 (5)	3 (6)
Black	7 (16)	6 (15)	12 (21)	11 (20)	15 (23)	14 (21)
Hispanic	1 (3)	2 (4)	2 (3)	2 (3)	2 (2)	2 (3)
White	88 (17)	87 (18)	81 (23)	82 (22)	76 (25)	77 (23)
Others^b^	3 (5)	4 (6)	3 (7)	4 (7)	4 (8)	5 (8)
Rural, %^c^	40 (40)	43 (40)	20 (32)	21 (32)	13 (26)	16 (28)
Medicaid eligible, %	13 (17)	13 (18)	16 (19)	16 (20)	20 (24)	19 (24)
HCC RAF score	0.9 (0.6)	0.7 (0.6)	1.0 (0.6)	1.0 (0.6)	1.0 (0.5)	1.0 (0.5)
HCC RAF score squared	2.3 (2.8)	1.9 (2.7)	2.8 (2.9)	2.9 (3.2)	2.8 (2.5)	2.9 (2.7)

Abbreviations: HCC, Hierarchical Condition Categories; RAF risk adjustment factor.

^a^ Reported as proportion of beneficiaries at the primary care practice level.

^b^ Includes self-reported race and ethnicity responses that are not White, Black, Hispanic, or Asian from the Medicare Master Beneficiary Summary File.

^c^ Rurality was defined at the beneficiary zip code level based on the Department of Agriculture’s Rural-Urban Commuting Area Codes.^22^

### Changes in Telehealth and In-Person Visit Volume

In 2019, the mean (SD) rate of telehealth visits was 7 (29), 5 (39), and 13 (115) visits per 1000 beneficiaries for low-, medium-, and high-tertile groups of telehealth use, respectively. In 2022, these mean (SD) rates increased to 194 (155), 654 (116), and 1469 (522) visits per 1000 beneficiaries for low-, medium-, and high-tertile groups of telehealth use, respectively (Table 1).

In 2019, the mean (SD) rate of in-person visits was 10 200 (6568), 12 781 (6534), and 16 183 (11 822) visits per 1000 beneficiaries for low-, medium-, and high-tertile groups of telehealth use, respectively. In 2022, these rates decreased to 7862 (9664), 11 670 (4921), and 13 557 (7492) visits per 1000 beneficiaries for low-, medium-, and high-tertile groups of telehealth use, respectively (Table 1).

### Changes in Low-Value Care Services Over Time and by Practice-Level Telehealth Use

#### Office-Based Services

Rates of cervical cancer screening for women older than 65 years decreased from 2019 to 2022 among all tertile groups of practice-level telehealth use (Table 2). Rates of low-value cervical cancer screening declined more from 2019 to 2022 in practices with medium (AME, −2.2; 95% CI, −4.2 to −0.3 services per 1000 beneficiaries) and high (AME, −2.9; 95% CI, −5.3 to −0.4 services per 1000 beneficiaries) telehealth use than those with low use, holding constant the distribution of practice-level covariates. (Table 2).

**Table 2.** Difference-in-Differences Estimates for the Association of Practice-Level Telehealth Use With Rates of Low-Value Care Services^a^

Low-value care	Services provided per 1000 beneficiaries			
	Prepandemic period (2019)	Postpandemic period (2022)	Difference	Difference in differences (95% CI)
**Office-based low-value services**				
Cervical cancer screening for women >65 y				
Low (reference)	13.1	10.1	−2.9	NA
Medium	12.9	7.7	−5.2	−2.2 (−4.2 to −0.3)
High	14.2	8.4	−5.8	−2.9 (−5.3 to −0.4)
**Mixed-modality low-value services**				
Colorectal cancer screening for patients >85 y				
Low (reference)	7.3	4.7	−2.6	NA
Medium	8.3	5.0	−3.4	−0.8 (−2.5 to 1.0)
High	7.9	6.5	−1.4	1.2 (−0.6 to 2.9)
**Laboratory-based low-value services**				
PSA testing for men >75 y				
Low (reference)	310	322	12	NA
Medium	311	312	1	−11 (−34 to 13)
High	321	339	18	6 (−18 to 31)
Total or free T3 level testing for patients with hypothyroidism				
Low (reference)	199	254	55	NA
Medium	165	163	−2	−57 (−88 to −26)
High	189	205	15	−40 (−70 to −9)
Vitamin D testing in the absence of kidney disease or hypercalcemia				
Low (reference)	0.53	0.19	−0.34	NA
Medium	0.76	0.25	−0.51	−0.17 (−0.44 to 0.09)
High	0.73	0.20	−0.53	−0.19 (−0.46 to 0.08)
**Imaging-based low-value services**				
CT of sinuses for uncomplicated acute rhinosinusitis				
Low (reference)	15.8	20.2	4.4	NA
Medium	12.9	19.9	7.0	2.6 (−1.9 to 7.2)
High	15.4	20.8	5.4	1.0 (−4.1 to 6.0)
Head imaging for uncomplicated headache				
Low (reference)	164	159	−5	NA
Medium	167	156	−11	−6 (−20 to 7)
High	162	144	−18	−13 (−28 to 2)
Back imaging for nonspecific low back pain				
Low (reference)	98	100	2	NA
Medium	100	101	1	−1 (−7 to 5)
High	95	93	−2	−4 (−10 to 2)

Abbreviations: PSA, prostate-specific antigen; CT, computed tomography; NA, not applicable.

^a^ Estimated using average marginal effects and reported in services per 1000 beneficiaries. Models adjust for practice-level characteristics, including beneficiary count, number of in-person outpatient visits per 1000 beneficiaries, age, gender, race and ethnicity, Medicaid dual-eligibility, and rurality.^22^ Risk adjustment was performed using the Centers for Medicare & Medicaid Services Hierarchical Condition Categories risk adjustment model to calculate average risk score and its square, both of which were included in our statistical model.^8,9,23^

#### Mixed-Modality Services

Rates of colorectal cancer screening for adults older than age 85 years decreased from 2019 to 2022 among all tertile groups of practice-level telehealth use (Table 2). There was no association between practice-level telehealth use and rates of low-value colorectal cancer screening.

#### Laboratory-Based Services

Changes in the rates of laboratory-based low-value care services varied. For low-value PSA and vitamin D testing, rates of these services either modestly increased or had similar rates between 2019 and 2022 across all levels of practice-level telehealth use (Table 2). For low-value thyroid testing, these rates decreased between 2019 and 2022 across all tertiles of practice-level telehealth use. Rates of low-value thyroid testing declined more from 2019 to 2022 in practices with medium (AME, −57; 95% CI, −88 to −26 services per 1000 beneficiaries) and high (AME, −40; 95% CI, −70 to −9 services per 1000 beneficiaries) telehealth use than those with low use, holding constant the distribution of practice-level covariates (Table 2).

#### Imaging-Based Services

Changes in the rates of imaging-based low-value care services varied. Rates of CT sinus imaging increased between 2019 and 2022 across all tertiles of practice-level telehealth use. There was a small decrease in rates of head imaging for uncomplicated headache while rates for low-value imaging for low back pain were largely unchanged over time. There was no association between practice-level telehealth use and rates of imaging-based low-value care services (Table 2). Full regression results of all low-value care outcomes are available in eTables 3 to 5 in Supplement 1.

## Discussion

Among Medicare fee-for-service beneficiaries who received care from primary care practices in Michigan, increased telehealth use was not associated with changes in rates for most low-value care services. For low-value cervical cancer screening and low-value thyroid testing, increased telehealth use was associated with decreased rates of these services. Collectively, these findings suggest that telehealth could be used to deliver primary care services without introducing wasteful or unnecessary care and may even help reduce office-based low-value care.

To our knowledge, this is the first study analyzing the association of telehealth use with low-value care services in primary care. Prior studies have investigated changes in high- and low-value care during the COVID-19 pandemic^24,25^; however, they did not study the impact of telehealth use. Other studies have examined the association of telehealth use and quality of care, specifically focusing on outcomes of hospitalizations and emergency department visits. An analysis by the Medicare Payment Advisory Commission found that high telehealth intensity in Hospital Service Areas (HSAs) was associated with an increase in ambulatory care–sensitive hospitalizations, relative to low telehealth intensity HSAs among fee-for-service Medicare beneficiaries.^8,9^ Similarly, Li et al^7^ found that high vs low practice-level telehealth use was associated with a small increase in ambulatory care–sensitive condition visits in a commercially insured population.

While these studies found that increased telehealth use was associated with more ambulatory care–sensitive hospitalizations, an important limitation acknowledged by both studies is the short analytic time frame that was limited to the initial COVID-19 pandemic onset and subsequent surges. Our results may differ from previous findings due to our longer study period that includes less COVID-19–related illness, which may be more reflective of prepandemic health behaviors. Additionally, we chose measures of low-value care as our primary outcome, which correlate with process measures rather than outcome measures. Given that process measures may be more sensitive of ambulatory quality compared with outcome measures,^26,27^ our findings suggest that higher practice-level telehealth use is not associated with decreased quality of care in the primary care setting.

Our findings are consistent with current literature on the association of telehealth with diagnostic testing. In a recent study examining the impact of telehealth on utilization and quality among Medicare fee-for-service beneficiaries, there were no significant changes in imaging services or laboratory testing between health systems with high and low levels of telehealth use.^10^ Our findings directly build on this work by focusing on the subset of diagnostic testing deemed to be unnecessary or wasteful diagnostic services, ie, low-value care. Our findings of no association between high practice-level telehealth use and changes in rates of most low-value diagnostic services may reassure policymakers concerned about telehealth encouraging unnecessary or wasteful testing due to added convenience.

The decreased rates of low-value cervical cancer screening with higher practice-level telehealth use may be explained by decreased rates of in-person visits from 2019 to 2022. As we measured rates of low-value cervical cancer screening through Papanicolaou testing, an office-based procedure, it is not surprising that fewer in-person visits limit the opportunity to deliver such services. These findings are consistent with recent evidence demonstrating lower rates of Papanicolaou testing overall with higher practice-level telehealth utilization among Medicare beneficiaries, although the study did not distinguish between high- and low-value cervical cancer screening.^10^ Potential explanations for our other significant finding of decreased rates in low-value thyroid testing with higher practice-level telehealth use are less obvious. We hypothesized that increased telehealth use may limit opportunities for individuals to undergo laboratory testing in conjunction with their in-person visit due to convenience. While we observed this pattern for low-value thyroid testing, we did not find an association with practice-level telehealth use among other laboratory-based low-value care services.

We should not underestimate the quality, equity, and cost implications of reductions in office-based low-value care services. Prior work has shown that many office-based low-value services, such as annual resting electrocardiograms, are common and contribute to large amounts of unnecessary spending.^28,29^ Additionally, there has been concern that minoritized populations disproportionately receive more low-value care services compared with their White counterparts.^30^ We acknowledge our study did not quantify high-value care and further research is needed to investigate whether high-value care may be reduced in similar ways low-value care was reduced with high practice-level telehealth use; however, our findings suggest the potential for high practice-level telehealth use to reduce office-based low-value care services.

### Limitations

Our study has several limitations. While we focused on several low-value services relevant to primary care, we were not able to comprehensively examine all low-value services, including low-value medication prescriptions, which may be differentially affected by telehealth use. Our study was performed among Medicare fee-for-service beneficiaries with a Michigan residence and may not be generalizable to the broader Medicare beneficiary population. Administrative claims data do not include clinical information, which limits our ability to measure overall quality of care. However, we used well-established methods of identifying low-value care services in the fee-for-service Medicare population, which addresses an important dimension within quality of care. Our study defined telehealth use at the practice level and we did not assess individual outcomes. However, because telehealth implementation often occurs at the organization level, our findings may be informative for policymakers and health systems. Additional research is needed at a national level to determine the impact of telehealth on low-value care services in primary care.

## Conclusions

In this cohort study of Medicare fee-for-service beneficiaries who received care from primary care practices in Michigan, increased telehealth use was not associated with changes in rates of most low-value care services. While the rapid growth of telehealth has enhanced access to care for individuals, it has also raised concern for unintended consequences in the form of wasteful or unnecessary care, ie, low-value care. Our study suggests that increased practice-level telehealth use was not associated with the delivery of low-value care services in primary care and may even help reduce office-based low-value care. As policymakers consider how telehealth impacts both the quality and cost of care, our findings can help inform policy that determines ongoing and future implementation of telehealth in Medicare.

## Notes

Supplement 1. **eTable 1.** Detail on Telehealth Services **eTable 2.** Detail on Low-Value Care Measures **eFigure.** Parallel Trends Assessment for Low-Value Care Services During Preintervention Period **eTable 3.** Multivariable Regression Results of Association Between Telehealth Use Tertile and Office-Based and Mixed-Modality Low-Value Services **eTable 4.** Multivariable Regression Results of Association Between Telehealth Use Tertile and Laboratory-Based Low-Value Services **eTable 5.** Multivariable Regression Results of Association Between Telehealth Use Tertile and Imaging-Based Low-Value Services
eReferences.

Supplement 2.
Data Sharing Statement

## References

[1] Wharton GA, Sood HS, Sissons A, Mossialos E. Virtual primary care: fragmentation or integration? Lancet Digit Health. 2019;1(7):e330-e331. doi:10.1016/S2589-7500(19)30152-9 33323207

[2] Nitiema P. Telehealth before and during the COVID-19 pandemic: analysis of health care workers’ opinions. J Med Internet Res. 2022;24(2):e29519. doi:10.2196/29519 34978532 PMC8887560

[3] Frank CJ, Lin LA. Defining and supporting high-quality telehealth for patients with opioid use disorder: the promise and potential pitfalls of telehealth expansion. Subst Abus. 2022;43(1):1370-1373. doi:10.1080/08897077.2022.2127140 36222798

[4] Sinsky CA, Jerzak JT, Hopkins KD. Telemedicine and team-based care: the perils and the promise. Mayo Clin Proc. 2021;96(2):429-437. doi:10.1016/j.mayocp.2020.11.020 33549262 PMC7857703

[5] Khoong EC, Sharma AE, Gupta K, Adler-Milstein J, Sarkar U. The abrupt expansion of ambulatory telemedicine: implications for patient safety. J Gen Intern Med. 2022;37(5):1270-1274. doi:10.1007/s11606-021-07329-9 35048294 PMC8768444

[6] O’Reilly-Jacob M, Mohr P, Ellen M, . Digital health & low-value care. Healthc (Amst). 2021;9(2):100533. doi:10.1016/j.hjdsi.2021.100533 33714891 PMC9342901

[7] Li KY, Ng S, Zhu Z, McCullough JS, Kocher KE, Ellimoottil C. Association between primary care practice telehealth use and acute care visits for ambulatory care-sensitive conditions during COVID-19. JAMA Netw Open. 2022;5(3):e225484. doi:10.1001/jamanetworkopen.2022.5484 35357448 PMC8972029

[8] Saharkhiz M, Rao T, Parker-Lue S, Borelli S, Johnson K, Cataife G. Telehealth expansion and Medicare beneficiaries’ care quality and access. JAMA Netw Open. 2024;7(5):e2411006. doi:10.1001/jamanetworkopen.2024.11006 38739388 PMC11091757

[9] MedPAC. Telehealth in Medicare. June 2023. Accessed September 27, 2023. https://www.medpac.gov/document/chapter-7-mandated-report-telehealth-in-medicare-june-2023-report/

[10] Nakamoto CH, Cutler DM, Beaulieu ND, Uscher-Pines L, Mehrotra A. The impact of telemedicine on Medicare utilization, spending, and quality, 2019-22. Health Aff (Millwood). 2024;43(5):691-700. doi:10.1377/hlthaff.2023.01142 38630943 PMC13400057

[11] Oakes AH, Radomski TR. Reducing low-value care and improving health care value. JAMA. 2021;325(17):1715-1716. doi:10.1001/jama.2021.3308 33830184

[12] Center for Value-Based Insurance Design. Reducing the utilization of low-value care. Accessed April 1, 2024. https://vbidcenter.org/initiatives/low-value-care/

[13] Cutter C, Berlin NL, Fendrick AM. Establishing a value-based ‘new normal’ for telehealth. Health Aff Forefront. Published online October 8, 2020. doi:10.1377/forefront.20201006.638022

[14] Schwartz AL, Landon BE, Elshaug AG, Chernew ME, McWilliams JM. Measuring low-value care in Medicare. JAMA Intern Med. 2014;174(7):1067-1076. doi:10.1001/jamainternmed.2014.1541 24819824 PMC4241845

[15] Schwartz AL, Jena AB, Zaslavsky AM, McWilliams JM. Analysis of physician variation in provision of low-value services. JAMA Intern Med. 2019;179(1):16-25. doi:10.1001/jamainternmed.2018.5086 30508010 PMC6583417

[16] Schwartz AL, Zaslavsky AM, Landon BE, Chernew ME, McWilliams JM. Low-value service use in provider organizations. Health Serv Res. 2018;53(1):87-119. doi:10.1111/1475-6773.12597 27861838 PMC5785325

[17] Boudreau E, Schwartz R, Schwartz AL, . Comparison of low-value services among Medicare Advantage and traditional Medicare beneficiaries. JAMA Health Forum. 2022;3(9):e222935. doi:10.1001/jamahealthforum.2022.2935 36218933 PMC9463603

[18] Pickering AN, Zhao X, Sileanu FE, . Assessment of care cascades following low-value prostate-specific antigen testing among veterans dually enrolled in the US Veterans Health Administration and Medicare systems. JAMA Netw Open. 2022;5(12):e2247180. doi:10.1001/jamanetworkopen.2022.47180 36520431 PMC9856440

[19] Shrank WH, Rogstad TL, Parekh N. Waste in the US health care system: estimated costs and potential for savings. JAMA. 2019;322(15):1501-1509. doi:10.1001/jama.2019.13978 31589283

[20] Centers for Medicare & Medicaid Services. Two-step attribution for claims-based quality outcome measures and per capita cost measures included in the value modifier. August 1, 2017. Accessed October 1, 2024. https://www.cms.gov/Medicare/Medicare-Fee-for-Service-Payment/PhysicianFeedbackProgram/Downloads/Attribution-Fact-Sheet.pdf

[21] Andino JJ, Zhu Z, Surapaneni M, Dunn RL, Ellimoottil C. Interstate telehealth use by Medicare beneficiaries before and after COVID-19 licensure waivers, 2017-20. Health Aff (Millwood). 2022;41(6):838-845. doi:10.1377/hlthaff.2021.01825 35666968

[22] Department of Agriculture, Economic Research Service. Rural-Urban Commuting Area Codes. Accessed May 10, 2024. https://www.ers.usda.gov/data-products/rural-urban-commuting-area-codes/

[23] Centers for Medicare & Medicaid Services. Risk adjustment. February 16, 2024. Accessed April 2, 2024. https://www.cms.gov/medicare/payment/medicare-advantage-rates-statistics/risk-adjustment

[24] Levine DM, Samal L, Neville BA, . The association of the first surge of the COVID-19 pandemic with the high- and low-value outpatient care delivered to adults in the USA. J Gen Intern Med. 2022;37(15):3979-3988. doi:10.1007/s11606-022-07757-1 36002691 PMC9400559

[25] Shahzad M, Zirui Song MD, Chernew M, Fendrick A. Changes in Use of Low-Value Services During the COVID-19 Pandemic. AJMC. April 22, 2022. Accessed November 14, 2023. https://www.ajmc.com/view/changes-in-use-of-low-value-services-during-the-covid-19-pandemic 10.37765/ajmc.2022.89031 36374618

[26] Mant J. Process versus outcome indicators in the assessment of quality of health care. Int J Qual Health Care. 2001;13(6):475-480. doi:10.1093/intqhc/13.6.475 11769750

[27] Birkmeyer JD, Kerr EA, Dimick JB. Improving the quality of quality measurement. In: Institute of Medicine, ed. *Performance Measurement: Accelerating Improvement*. The National Academies Press; 2006:177-203. doi:10.17226/11517

[28] Oronce CIA, Fendrick AM, Ladapo JA, Sarkisian C, Mafi JN. The utilization and costs of grade D USPSTF services in Medicare, 2007-2016. J Gen Intern Med. 2021;36(12):3711-3718. doi:10.1007/s11606-021-06784-8 33852141 PMC8045442

[29] Chernew M, Fendrick AM, Fischer K. Utilization and spending on low-value medical care across four states. V-BID Health. May 2022. Accessed October 1, 2024. https://vbidhealth.com/wp-content/uploads/2022/05/Utilization-and-Spending-on-Low-Value-Medical-Care-Across-Four-States-VOL2.pdf

[30] Schpero WL, Morden NE, Sequist TD, Rosenthal MB, Gottlieb DJ, Colla CH. For selected services, Blacks And Hispanics more likely to receive low-value care than Whites. Health Aff (Millwood). 2017;36(6):1065-1069. doi:10.1377/hlthaff.2016.1416 28583965 PMC5568010

