# Supplementary material for: Primary Care Practice Telehealth Use and Low-Value Care Services
Source: JAMA Netw Open. 2024 Nov 7;7(11):e2445436. doi: 10.1001/jamanetworkopen.2024.45436 (PMC11544489; doi:10.1001/jamanetworkopen.2024.45436)
Supplement: Supplement 1. — eTable 1. Detail on Telehealth Services eTable 2. Detail on Low-Value Care Measures eFigure. Parallel Trends Assessment for Low-Value Care Services During Preintervention Period eTable 3. Multivariable Regression Results of Association Between Telehealth Use Tertile and Office-Based and Mixed-Modality Low-Value Services eTable 4. Multivariable Regression Results of Association Between Telehealth Use Tertile and Laboratory-Based Low-Value Services eTable 5. Multivariable Regression Results of Association Between Telehealth Use Tertile and Imaging-Based Low-Value Services eReferences. [file jamanetwopen-e2445436-s001.pdf]

Supplemental Online Content

Liu T, Zhu Z, Thompson MP, et al. Primary care practice telehealth use and low-value care services. *JAMA Netw Open.* 2024;7(11):e2445436. doi:10.1001/jamanetworkopen.2024.45436

eTable 1. Detail on Telehealth Services

eTable 2. Detail on Low-Value Care Measures

eFigure. Parallel Trends Assessment for Low-Value Care Services During Preintervention Period

eTable 3. Multivariable Regression Results of Association Between Telehealth Use Tertile and Office-Based and Mixed-Modality Low-Value Services

eTable 4. Multivariable Regression Results of Association Between Telehealth Use Tertile and Laboratory-Based Low-Value Services

eTable 5. Multivariable Regression Results of Association Between Telehealth Use Tertile and Imaging-Based Low-Value Services

eReferences.

This supplemental material has been provided by the authors to give readers additional information about their work.

© 2024 Liu T et al. *JAMA Network Open.*

eTable 1. Detail on Telehealth Services

Type of telehealth	Healthcare Common Procedure Coding System (HCPCS) codes
Modifier Codes/Place of Service (POS)	Modifier: G0, GQ, GT, FR, FQ, 93, 95<br><br>Place of service: 02, 10
Video/Audio	In addition of having modifier/POS, we used the following HCPCS codes in CMS annual telehealth list[1]:<br><br>0362T, 0373T, 77427, 90875, 90901, 90951, 90952, 90953, 90954, 90955, 90956, 90957, 90958, 90959, 90960, 90961, 90962, 90963, 90964, 90965, 90966, 90967, 90968, 90969, 90970, 92002, 92004, 92012, 92014, 92526, 92550, 92552, 92553, 92555, 92556, 92557, 92563, 92565, 92567, 92568, 92570, 92587, 92588, 92601, 92602, 92603, 92604, 92607, 92608, 92609, 92610, 92625, 92626, 92627, 93750, 93797, 93798, 94002, 94003, 94004, 94005, 94625, 94626, 94664, 95970, 95971, 95972, 95983, 95984, 96105, 96110, 96112, 96113, 96125, 96170, 96171, 97110, 97112, 97116, 97129, 97130, 97150, 97151, 97152, 97153, 97154, 97155, 97156, 97157, 97158, 97161, 97162, 97163, 97164, 97165, 97166, 97167, 97168, 97530, 97537, 97542, 97750, 97755, 97760, 97761, 97763, 98960, 98961, 98962, 99202, 99203, 99204, 99205, 99211, 99212, 99213, 99214, 99215, 99221, 99222, 99223, 99231, 99232, 99233, 99234, 99235, 99236, 99238, 99239, 99281, 99282, 99283, 99284, 99285, 99291, 99292, 99304, 99305, 99306, 99307, 99308, 99309, 99310, 99315, 99316, 99341, 99342, 99344, 99345, 99347, 99348, 99349, 99350, 99468, 99469, 99471, 99472, 99473, 99475, 99476, 99477, 99478, 99479, 99480, 99483, 99495, 99496, G0136, G0316, G0317, G0318, G0410, G0422, G0423, G0508, G0509, G3002, G3003, G9685, 0591T, 0592T, 0593T, 90785, 90791, 90792, 90832, 90833, 90834, 90836, 90837, 90838, 90839, 90840, 90845, 90846, 90847, 90853, 92507, 92508, 92521, 92522, 92523, 92524, 96116, 96121, 96127, 96130, 96131, 96132, 96133, 96136, 96137, 96138, 96139, 96156, 96158, 96159, 96160, 96161, 96164, 96165, 96167, 96168, 97535, 97802, 97803, 97804, 98966, 98967, 98968, 99406, 99407, 99441, 99442, 99443, 99497, 99498, G0108, G0109, G0270, G0296, G0396, G0397, G0406, G0407, G0408, G0420, G0421, G0425, G0426, G0427, G0438, G0439, G0442, G0443, G0444, G0445, G0446, G0447, G0459, G0506, G0513, G0514, G2086, G2087, G2088, G2211, G2212
Audio Only	In addition of having modifier/POS, we used the following HCPCS codes in CMS annual telehealth list[1]:<br><br>0591T, 0592T, 0593T, 90785, 90791, 90792, 90832, 90833, 90834, 90836, 90837, 90838, 90839, 90840, 90845, 90846, 90847, 90853, 92507, 92508, 92521, 92522, 92523, 92524, 96116, 96121, 96127, 96130, 96131, 96132, 96133, 96136, 96137, 96138, 96139, 96156, 96158, 96159, 96160, 96161, 96164, 96165, 96167, 96168, 97535, 97802, 97803, 97804, 98966, 98967, 98968, 99406, 99407, 99441, 99442, 99443, 99497, 99498, G0108, G0109, G0270, G0296, G0396, G0397, G0406, G0407, G0408, G0420, G0421, G0425, G0426, G0427, G0438, G0439, G0442, G0443, G0444, G0445, G0446, G0447, G0459, G0506, G0513, G0514, G2086, G2087, G2088, G2211, G2212
Phone	99441, 99442, 99443
Online digital E&M for established patients	99421, 99422, 99423, G2061/98970, G2062/98971, G2063/98972
Remote physiologic monitoring	99453, 99454, 99457, 99458, 99091
Remote therapeutic monitoring	98975, 98976, 98977, 98980, 98981
Interprofessional internet consultation	99446, 99447, 99448, 99449, 99451, 99452
Virtual check-ins	G2010/G2250, G2012/G2251, G2252
Safety-net providers	G0071, G2025
Other types of telehealth	G0459, 98966, 98967, 98968, 98969, 99444, 0448T, 994X0, G0320, G0321, G0322

© 2024 Liu T et al. *JAMA Network Open.*

eTable 2. Detail on Low-Value Care Measures

Measure	Codes for Detection and Exclusion[a]	Eligible Population[b]
**Office-based**		
Cervical cancer screening for women over age 65	Detection: CPT: G0101 G0123 G0124 G0141 G0143 G0144 G0145 G0147 G0148 P3000 P3001 Q0091 (cervical screening)<br><br>Exclusion: ICD-10: Z8540 Z8541 Z8544 (history of cervical cancer)	Women over 65
**Mixed-modality**		
Colorectal cancer screening for patients over age 85	Detection: CPT: 45330-45350 45378-45392 G0104-G0106 G0120-G0122 G0328 82270 45398 (sigmoidoscopy, colonoscopy, barium enema or blood occult test for colon cancer screening) Z1211 (colon screening)	Patients over 85
**Lab-based**		
Prostate-specific antigen (PSA) testing for men over age 75	Detection: CPT/HCPCS: G0103 (PSA screening)	Men Over 75
Total or free T3 level testing for patients with hypothyroidism	Detection: CPT: 84480 84481 (total or free T3). CCW: Hypothyroidism first indication date	Patients with hypothyroidism
1,25-dihydroxyvitamin D testing in the absence of hypercalcemia or decreased kidney function	Detection: CPT:82652 (1,25-dihydroxyvitamin D3)<br><br>Exclusion: CCW: Chronic kidney disease first indication date<br>ICD-10: E83.52 (Hypercalcemia) N25.81 (Secondary hyperparathyroidism of renal origin), D86 A15 A17 A18 A19<br>C44 C50 C64 C65 C67 C56 C81-C96 D45 (sarcoidosis, TB, select neoplasms, polycythemia)<br>ICD-9: 27542 (hypercalcemia) 58881 (secondary hyperparathyroidism of renal origin) 135x 01x 173x 174x 175x 1890 1891 188x 1830 200x-208x (sarcoidosis, TB, select neoplasms)	All patients
**Imaging-based**		
CT of the sinuses for uncomplicated acute rhinosinusitis	Detection: CPT: 70486-70488 (CT of maxillofacial area) ICD-10: J01 J32 (sinusitis)<br><br>Exclusion: E84 (cystic fibrosis) B20 B9735 D80-D84 D89 (immune disorders) J33 (nasal polyp) H00xx H01xx H05xx (eyelid/orbit inflammation) S0xxx S1xxx (head or face trauma)	Patients with sinusitis diagnosis
Head imaging for uncomplicated headache	Detection: CPT: 70450 70460 70470 70551-70553 (CT or MRI of head or brain) ICD-10: R51 G43 G44 (headache/migraine)<br><br>Exclusion: ICD-10: G443 G4453 (post-traumatic or thunderclap headache) G434 (hemiplegic migraine) G46 (vascular syndromes of brain in cerebrovascular diseases) M315 M316 (giant cell arteritis) G40 R56 (epilepsy or convulsions) I6xxx G45 G46 (cerebrovascular diseases) S0xxx S1xxx (head or face trauma) R20 R25-R27 R290xx R291xx R292xx R29701-R2970 R2971- R2974 R2981 R2990 R40 R41 R43 R47 R930 (altered mental status, nervous and musculoskeletal system symptoms, including gait abnormality, meningismus, disturbed skin sensation, speech deficits) Z8673 (personal history of stroke/TIA) Z85 (personal history of malignant neoplasm)	Patients with headache diagnosis
Back imaging for patients with nonspecific low back pain	Detection: CPT: 72010 72020 72052 72100 72110 72114 72120 72200 72202 72220 72131-72133 72141 72142 72146-72149 72156 72157 72158 (radiologic, CT, and MRI imaging of spine) ICD-10: M4720 M4726-M4728 M47816-M47819 M47896-M47899 M479 M4806-M4808 M5116 M5117 M5126 M5127 M5136 M5137 M5186 M5186 M5187 M519 M532C6 M532X7 M532X8 M533 M5386-M5388 M5416- M5418 M5430-M5432 M5440-M5442 M545 M5489 M549 M9903 M9904 M9923 M9933 M9943 M9953 M9963 M973 M9983 M9984 S335 S336 S338 S339 S39002 S39012 S39092 S3982 S3992 (back pain, various causes)	Patients with back pain

© 2024 Liu T et al. *JAMA Network Open.*

	Exclusion: ICD-10: Cxxxx D0 D37-D44 D46-D49 Z85 (neoplasms) S0xxx S10xx-S12xx S130x-S132x S140x S1411-S1415 S21xx S22xx S230x-S232x S240x S241x S26xx-S28xx S310x S32xx S330x-S334x S340x S3411-S3413 S36xx-S38xx S42xx S430x-S433x S490x S491x S52xx S530x-S533x S57xx S58xx S590x-S592x S62xx S630x-S634x S72xx S730x S77xx S78xx S790x S791x S82xx S830x S831x S87xx S88xx S890x-S893x S92xx S9030x S931x S97xx S98xx S990x-S992x T79xx (trauma) A15 A17-A19 (tuberculosis) F11 F13-F15 (drug abuse) M462-M465 (osteomyelitis) G061 G834 (neurologic impairment) I33 I38 I39 (endocarditis) A40 A41 (septicemia) R50 R6883 R630 R634 R636 R538 R61 D649 (fever, weight loss, malaise, night sweats, anemia unspecified)	

[a]Defined according to taxonomy from Schwartz et al 2014.[2]
[b]Defined according to taxonomy from Schwartz et al 2019.[3]

© 2024 Liu T et al. *JAMA Network Open.*

eFigure. Parallel Trends Assessment for Low-Value Care Services During Preintervention Period

A. Cervical cancer screening for women over age 65

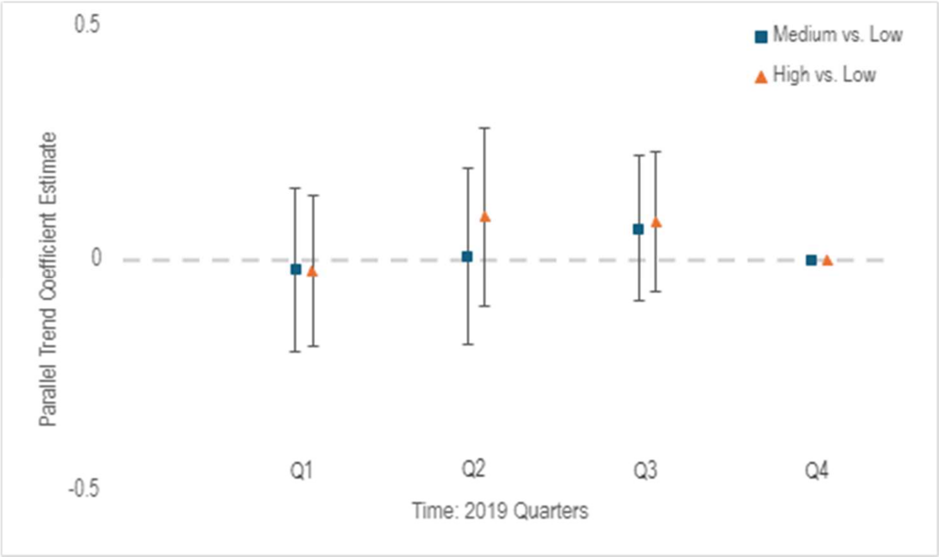

B. Colorectal cancer screening for adults over age 85

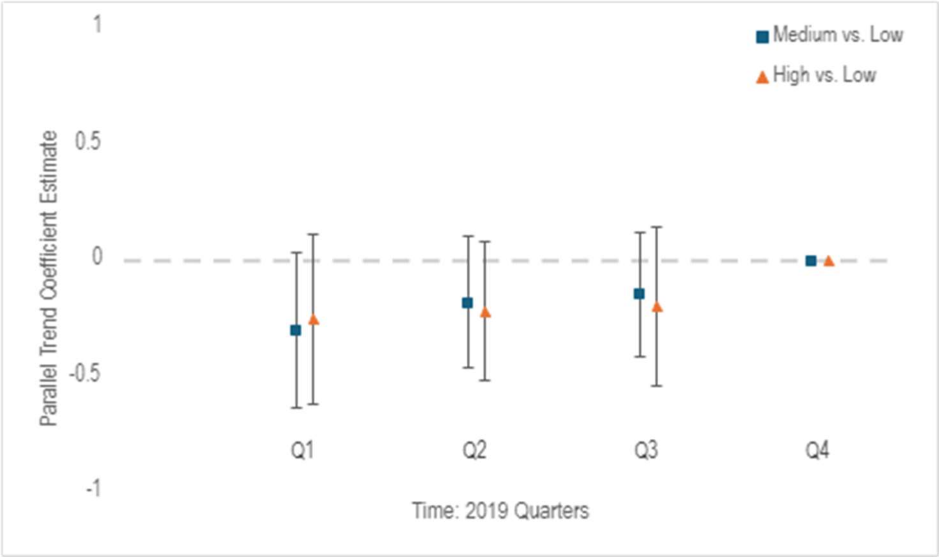

C. Prostate-specific antigen testing for men over age 75

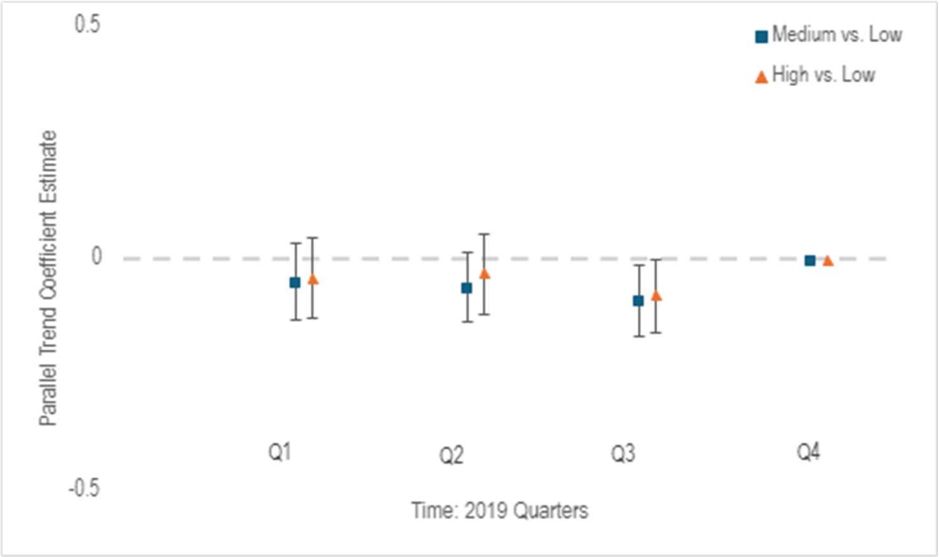

B. Total or free T3 level testing for patients with hypothyroidism

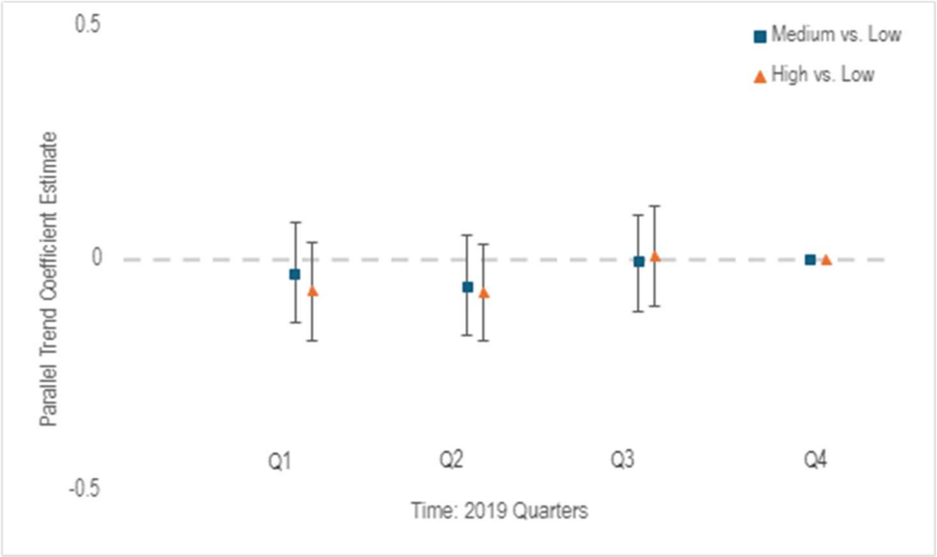

© 2024 Liu T et al. *JAMA Network Open.*

C.1,25-dihydroxyvitamin D testing in absence of hypercalcemia or decreased kidney function

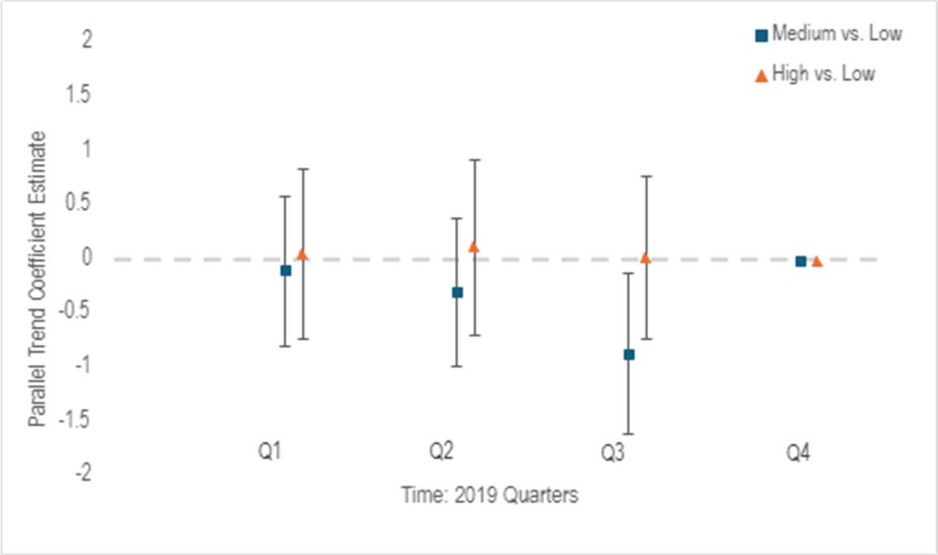

D. Computed tomography of sinuses for uncomplicated acute rhinosinusitis

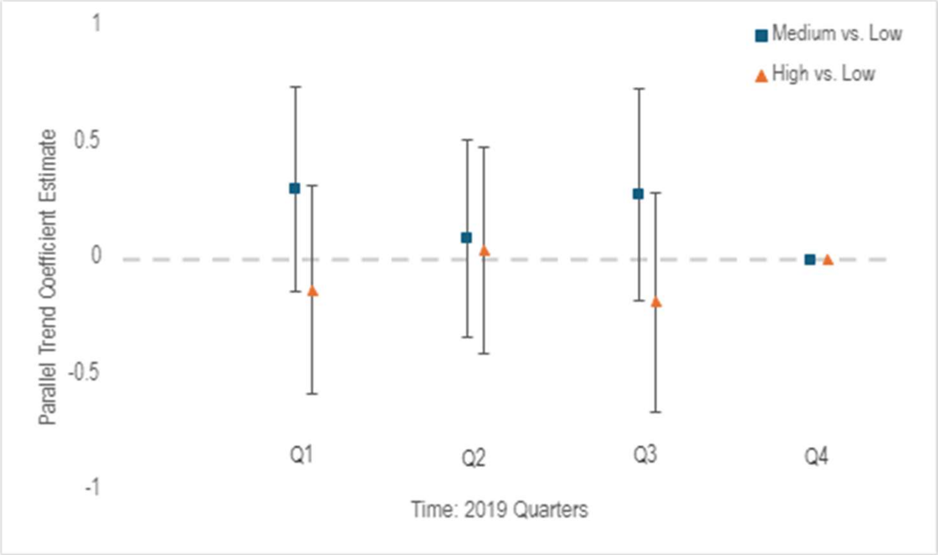

E. Head imaging for uncomplicated headache

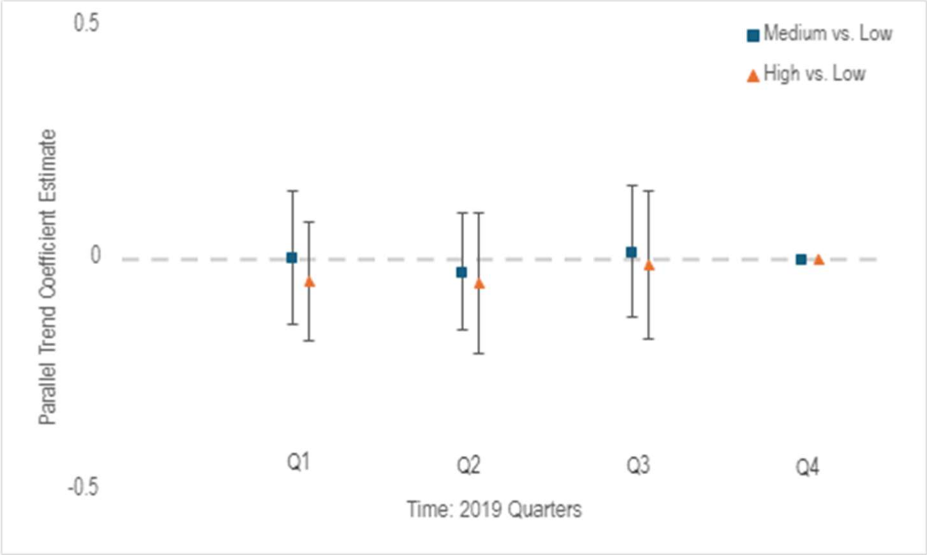

F. Back imaging for patients with non-specific low back pain

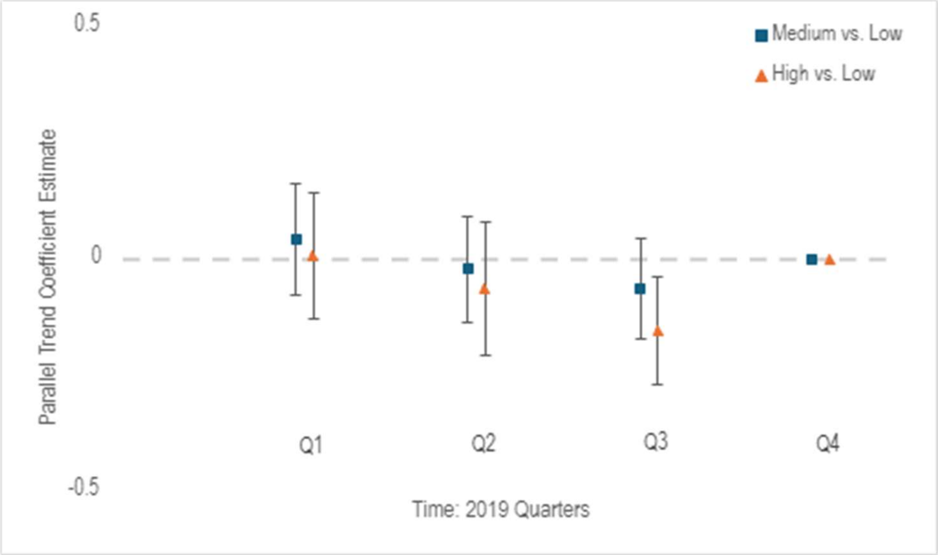

Note: Adjusted rates of low-values services were calculated in each quarter of 2019 to test the parallel trends assumption in the "pre-intervention" period. Adjusted rates of low-value services were calculated for high- and medium-telehealth use practices and compared to low-telehealth use practices. The y-axis represents the difference between rates of low-value services between high- and medium-telehealth use practices and those of low-telehealth use practices. The last quarter of 2019 was used as the reference period in these comparisons

© 2024 Liu T et al. *JAMA Network Open.*

eTable 3. Multivariable Regression Results of Association Between Telehealth Use Tertile and Office-Based and Mixed-Modality Low-Value Services

	**Office-based[a]**		**Mixed-modality[b]**	
**Variable**	**Estimated Coefficient (95% CI)**	**p value**	**Estimated Coefficient (95% CI)**	**p value**
TH tertile # period[c]				
Medium - post	-0.26 (-0.46, -0.07)	0.009	-0.08 (-0.32, 0.16)	0.511
High - post	-0.27 (-0.53, -0.01)	0.042	0.24 (-0.01, 0.49)	0.061
TH tertile				
Medium	-0.02 (-0.17, 0.14)	0.842	0.14 (-0.15, 0.43)	0.360
High	0.09 (-0.09, 0.26)	0.353	0.09 (-0.22, 0.39)	0.583
In-person tertile				
Medium	0.10 (-0.05, 0.26)	0.176	0.24 (-0.02, 0.49)	0.065
High	0.31 (0.12, 0.49)	0.001	0.53 (0.19, 0.86)	0.002
Period (ref = 2019)				
Post (2022)	-0.25 (-0.38, -0.13)	<0.001	-0.43 (-0.62, -0.25)	<0.001
Female Sex	0.39 (-0.57, 1.35)	0.426	0.59 (-0.38, 1.56)	0.237
Age (ref = ≥85)				
65 – 74	2.17 (1.29, 3.06)	<0.001	-2.58 (-3.58, -1.59)	<0.001
75 – 84	2.52 (1.21, 3.83)	<0.001	-0.17 (-1.76, 1.43)	0.839
Race/ethnicity (ref = other)				
White	-0.74 (-1.79, 0.31)	0.166	-1.04 (-2.56, 0.49)	0.183
Black	-1.01 (-2.09, 0.07)	0.067	-0.43 (-1.98, 1.12)	0.585
Hispanic	-8.77 (-12.93, -4.60)	<0.001	-2.26 (-8.89, 4.38)	0.505
Asian	-0.03 (-2.00, 1.93)	0.973	-4.33 (-8.90, 0.23)	0.063
Share of rural beneficiaries[d]	-0.11 (-0.29, 0.08)	0.255	0.23 (-0.15, 0.62)	0.231
Share of Medicaid-eligible beneficiaries	-0.91 (-1.48, -0.35)	0.001	-0.89 (-1.77, -0.02)	0.045
HCC RAF Score	-0.48 (-0.90, -0.06)	0.026	0.44 (-0.32, 1.20)	0.256
HCC RAF Score squared	0.05 (-0.03, 0.14)	0.214	-0.05 (-0.20, 0.09)	0.451

Abbreviations: TH, telehealth; HCC, Hierarchical Condition Categories; RAF, Risk Adjustment Factor

[a]Cervical cancer screening for women over age 65
[b]Colorectal cancer screening for patients over age 85
[c]Interaction between telehealth tertile (low, medium, high) and the time period (pre vs post)
[d]Rurality was defined at the beneficiary zip code level based on the Department of Agriculture's rural-urban commuting area codes[4]

© 2024 Liu T et al. *JAMA Network Open.*

eTable 4. Multivariable Regression Results of Association Between Telehealth Use Tertile and Laboratory-Based Low-Value Services

	PSA testing for men >75 y		Total or free T3 level testing for patients with hypothyroidism		Vitamin D testing in the absence of kidney disease or hypercalcemia	
**Variable**	**Coefficient Estimate (95% CI)**	**p value**	**Coefficient Estimate (95% CI)**	**p value**	**Coefficient Estimate (95% CI)**	**p value**
TH tertile # period[a]						
Medium - post	-0.03 (-0.11, 0.04)	0.379	-0.26 (-0.40, -0.11)	<0.001	-0.07 (-0.71, 0.57)	0.821
High - post	0.02 (-0.06, 0.09)	0.657	-0.17 (-0.30, -0.04)	0.013	-0.28 (-1.03, 0.48)	0.477
TH tertile						
Medium	0.00 (-0.08, 0.09)	0.970	-0.19 (-0.39, 0.02)	0.077	0.37 (-0.01, 0.75)	0.056
High	0.04 (-0.10, 0.18)	0.615	-0.05 (-0.22, 0.13)	0.590	0.32 (-0.06, 0.71)	0.102
In-person tertile						
Medium	0.31 (0.21, 0.42)	<0.001	-0.02 (-0.19, 0.15)	0.822	0.44 (0.04, 0.83)	0.032
High	0.54 (0.37, 0.70)	<0.001	0.32 (0.11, 0.53)	0.003	0.87 (0.36, 1.38)	0.001
Period (ref = 2019)						
Post (2022)	0.04 (-0.02, 0.10)	0.224	0.25 (0.14, 0.35)	<0.001	-1.02 (-1.50, -0.55)	<0.001
Female Sex	-0.26 (-0.71, 0.19)	0.252	-0.01 (-0.87, 0.85)	0.979	1.02 (-0.86, 2.91)	0.286
Age (ref = ≥85)						
65 – 74	0.85 (0.34, 1.37)	0.001	1.69 (0.80, 2.58)	<0.001	-1.05 (-3.40, 1.31)	0.382
75 – 84	2.91 (2.23, 3.59)	<0.001	1.57 (0.17, 2.98)	0.029	0.55 (-3.83, 4.92)	0.806
Race/ethnicity (ref = other)						
White	-1.08 (-1.79, -0.36)	0.003	-1.26 (-2.33, -0.18)	0.022	-3.02 (-5.26, -0.78)	0.008
Black	-0.80 (-1.49, -0.11)	0.023	-0.78 (-1.80, 0.24)	0.134	-1.58 (-3.94, 0.78)	0.188
Hispanic	-4.50 (-6.69, -2.31)	<0.001	-4.53 (-8.79, -0.27)	0.037	-2.53 (-8.64, 3.58)	0.417
Asian	-1.42 (-3.18, 0.33)	0.112	-2.91 (-5.73, -0.08)	0.043	-3.79 (-11.24, 3.66)	0.319
Share of rural beneficiaries[b]	-0.20 (-0.32, -0.08)	0.001	-0.59 (-0.82, -0.35)	<0.001	0.57 (-0.06, 1.20)	0.076
Share of Medicaid-eligible beneficiaries	0.03 (-0.29, 0.36)	0.841	0.34 (-0.11, 0.80)	0.133	0.39 (-0.79, 1.58)	0.517
HCC RAF Score	0.34 (0.07, 0.61)	0.012	0.31 (-0.23, 0.84)	0.261	0.24 (-0.89, 1.37)	0.675
HCC RAF Score Squared	-0.12 (-0.18, -0.07)	<0.001	-0.05 (-0.14, 0.04)	0.278	-0.03 (-0.22, 0.16)	0.764

Abbreviations: PSA, prostate-specific antigen; TH, telehealth; HCC, Hierarchical Condition Categories; RAF, Risk Adjustment Factor

[a]Interaction between telehealth tertile (low, medium, high) and the time period (pre vs post)
[b]Rurality was defined at the beneficiary zip code level based on the Department of Agriculture's rural-urban commuting area codes[4]

© 2024 Liu T et al. *JAMA Network Open.*

eTable 5. Multivariable Regression Results of Association Between Telehealth Use Tertile and Imaging-Based Low-Value Services

	CT of sinuses for uncomplicated acute rhinosinusitis		Head imaging for uncomplicated headache		Back imaging for non-specific low back pain	
**Variable**	**Coefficient Estimate (95% CI)**	**p value**	**Coefficient Estimate (95% CI)**	**p value**	**Coefficient Estimate (95% CI)**	**p value**
TH tertile # period[a]						
Medium - post	0.19 (-0.06, 0.44)	0.142	-0.04 (-0.12, 0.05)	0.379	-0.01 (-0.07, 0.05)	0.754
High - post	0.05 (-0.21, 0.32)	0.682	-0.09 (-0.18, 0.01)	0.072	-0.04 (-0.10, 0.02)	0.214
TH tertile						
Medium	-0.20 (-0.40, -0.01)	0.039	0.02 (-0.04, 0.08)	0.562	0.01 (-0.06, 0.09)	0.691
High	-0.03 (-0.22, 0.17)	0.799	-0.01 (-0.09, 0.07)	0.808	-0.03 (-0.10, 0.04)	0.402
In-person tertile						
Medium	-0.20 (-0.40, 0.00)	0.052	0.03 (-0.04, 0.09)	0.411	-0.03 (-0.10, 0.05)	0.444
High	-0.11 (-0.33, 0.11)	0.312	0.06 (0.00, 0.12)	0.063	-0.03 (-0.11, 0.06)	0.526
Period (ref = 2019)						
Post (2022)	0.24 (0.06, 0.43)	0.011	-0.03 (-0.09, 0.03)	0.289	0.02 (-0.03, 0.06)	0.519
Female Sex	-0.61 (-1.46, 0.23)	0.156	0.15 (-0.11, 0.40)	0.263	0.27 (0.05, 0.49)	0.015
Age (ref = ≥85)						
65 – 74	-0.53 (-1.63, 0.57)	0.347	-0.33 (-0.65, -0.01)	0.046	-0.11 (-0.38, 0.15)	0.399
75 – 84	-0.51 (-2.40, 1.37)	0.592	-0.04 (-0.58, 0.49)	0.872	0.25 (-0.17, 0.66)	0.241
Race/ethnicity (ref = other)						
White	-1.11 (-2.41, 0.19)	0.093	-0.03 (-0.51, 0.44)	0.891	-0.14 (-0.64, 0.36)	0.581
Black	-0.54 (-1.82, 0.75)	0.412	0.41 (-0.05, 0.87)	0.084	-0.41 (-0.93, 0.10)	0.113
Hispanic	1.26 (-1.22, 3.73)	0.320	-0.53 (-1.77, 0.71)	0.401	0.52 (-0.53, 1.58)	0.333
Asian	-2.29 (-4.85, 0.28)	0.081	0.27 (-0.62, 1.16)	0.557	-0.07 (-1.11, 0.97)	0.895
Share of rural beneficiaries[b]	-0.41 (-0.67, -0.14)	0.003	0.02 (-0.06, 0.10)	0.646	-0.15 (-0.21, -0.08)	<0.001
Share of Medicaid-eligible beneficiaries	-0.77 (-1.46, -0.09)	0.027	-0.29 (-0.47, -0.10)	0.002	-0.60 (-0.81, -0.39)	<0.001
HCC RAF Score	-0.04 (-0.73, 0.66)	0.916	0.48 (0.32, 0.64)	<0.001	-0.08 (-0.22, 0.07)	0.289
HCC RAF Score Squared	-0.02 (-0.14, 0.10)	0.754	-0.05 (-0.08, -0.02)	<0.001	0.04 (0.01, 0.06)	0.004

Abbreviations: CT, computed tomography; TH, telehealth; HCC, Hierarchical Condition Categories; RAF, Risk Adjustment Factor

[a]Interaction between telehealth tertile (low, medium, high) and the time period (pre vs post)
[b]Rurality was defined at the beneficiary zip code level based on the Department of Agriculture's rural-urban commuting area codes[4]

© 2024 Liu T et al. *JAMA Network Open.*

eReferences

1. Centers for Medicare & Medicaid Services. List of Telehealth Services. November 13, 2023. Accessed September 11, 2024. https://www.cms.gov/medicare/coverage/telehealth/list-services
2. Schwartz AL, Landon BE, Elshaug AG, Chernew ME, McWilliams JM. Measuring Low-Value Care in Medicare. JAMA Internal Medicine. 2014;174(7):1067-1076.
3. Schwartz AL, Jena AB, Zaslavsky AM, McWilliams JM. Analysis of Physician Variation in Provision of Low-Value Services. JAMA Internal Medicine. 2019;179(1):16-25.
4. Department of Agriculture, Economic Research Service. Rural-Urban Commuting Area Codes. Accessed May 10, 2024. https://www.ers.usda.gov/data-products/rural-urban-commuting-area-codes

© 2024 Liu T et al. *JAMA Network Open.*
