# Supplementary material for: Primary Care Practice Telehealth Use and Low-Value Care Services
Source: JAMA Netw Open. 2024 Nov 7;7(11):e2445436. doi: 10.1001/jamanetworkopen.2024.45436 (PMC11544489; doi:10.1001/jamanetworkopen.2024.45436)
Supplement: Supplement 2. — Data Sharing Statement [file jamanetwopen-e2445436-s002.pdf]

Data Sharing Statement

Liu. Primary Care Practice Telehealth Use and Low-Value Care Services. *JAMA Netw Open*. Published November 07, 2024. doi:10.1001/jamanetworkopen.2024.45436

Data
Data available: No

Additional Information
Explanation for why data not available: Our data use agreement with Centers for Medicare & Medicaid Services does not allow us to share the data.
